# The financial burden of juvenile idiopathic arthritis: a Nova Scotia experience

**DOI:** 10.1186/1546-0096-11-24

**Published:** 2013-05-29

**Authors:** Andrea Ens, Bianca Lang, Suzanne Ramsey, Elizabeth Stringer, Adam M Huber

**Affiliations:** 1 IWK Health Centre, Halifax, Nova Scotia, Canada; 2 Dalhousie University, Halifax, Nova Scotia, Canada

**Keywords:** Juvenile idiopathic arthritis, Financial burden, Cost

## Abstract

**Background:**

Juvenile idiopathic arthritis (JIA) is the most common childhood rheumatic illness. There is little published data on the financial burden of this illness. The primary objective of this study was to determine the annual costs borne by families of a child with JIA living in Nova Scotia (NS).

**Methods:**

All families in NS with a child followed in the Pediatric Rheumatology Clinic at the Izaak Walton Killam Health Centre (IWK) in 2009 were mailed a self-report questionnaire. The questionnaire evaluated disease related costs, gross household income and perceived financial burden. Dillman's method was used to optimize return rates. Descriptive statistics were used to summarize results. Spearman’s correlation coefficient was used to assess the relationship of distance from the IWK and cost. The Mann–Whitney U test was used to compare median costs between groups.

**Results:**

Of 172 possible respondents, we received 54 completed questionnaires and 11 blank questionnaires (overall response rate 31.4%). Approximately one third (35.9%) of parents rated the financial burden as moderate or large and 36% rated financial resources available as poor. The median annual total cost per patient was $619.50 CAD (range 0, $5535) which was a median 0.7% (range 0, 37%) of gross household incomes. The largest expense for families was visit related costs. There was not a significant relationship between total annual costs and distance from the IWK (r_s_ = 0.18, P = 0.2). Families of a child with oligoarthritis had significantly lower costs than the families of a child with another subtype of JIA ($359.00 CAD vs. $877.00 CAD, P = 0.02).

**Conclusions:**

The costs associated with having a child with JIA in NS are on average modest, but may be considerable for some families. Oligoarticular JIA is associated with smaller costs. Many families perceive the burden to be at least moderate and the availability of financial resources to be poor. Supports should be targeted to those families most in need.

## Background

Juvenile idiopathic arthritis (JIA) is the most common rheumatic illness affecting children with approximately 1 in 1000 children having one of 7 categories [1]. While there can be considerable variation in number of joints affected, disease severity and non-articular features, all children with JIA have a chronic illness which requires ongoing treatment and medical monitoring. This results in financial costs to families with JIA.

The Canadian health care system bears a significant portion of the medical costs, particularly physician and institution-based services. However, costs related to prescription medications, assistive devices and physiotherapy are often not included. Not all families have health insurance (whether private or government administered) to assist with these additional costs. There are also other non-medical costs which can add to the financial burden. These include costs associated with visits for medical care (travel, parking, meals, accommodations) and loss of paid work. The cost to families in order to provide their children with the recommended standard of care is unknown. However, anecdotal experience suggests that the burden for some families can be considerable.

The goal of this study was to determine the annual out-of-pocket medical and related non-medical expenses borne by Nova Scotian families who have a child with JIA. We also assessed perceptions of financial burden and financial support for these same families.

## Methods

### Study design and population

The Izaak Walton Killam Health Centre (IWK), located in Halifax, Nova Scotia, provides tertiary sub-specialist pediatric care to children in Nova Scotia (NS), New Brunswick and Prince Edward Island, Canada. This hospital is the only source of pediatric rheumatology care in these provinces, and most children with JIA are followed here.

A questionnaire was created to estimate the annual out-of-pocket costs borne by families as a result of their child’s JIA (Additional file 1: Appendix). To ensure content validity, members of the allied health care team and a convenience sample of selected families followed in the clinic reviewed the questionnaire. The questionnaire was mailed to all families in NS with a child followed in the Pediatric Rheumatology Clinic at the IWK for a diagnosis of JIA [1]. Families with more than one child affected by JIA were excluded as were children with JIA and another concurrent, unrelated chronic disease. A parent or primary caregiver was asked to complete the paper-based questionnaire and mail it back to the investigators in a pre-stamped envelope. The Dillman method was used to optimize response [2]. Specifically, all potential participants were mailed a card indicating that they would be receiving a questionnaire. The questionnaire was mailed 1 week later, with a reminder mailed 2 weeks after that. All mailings included a phone number for additional information or to opt out of future mailings. A chart review was also completed to document JIA category, medication use, number of appointments for clinics, medication infusions, and joint injections, and the number and duration of admissions.

Approval from the Research Ethics Board at the IWK was obtained. Return of the questionnaire constituted implied consent for participation in this research study.

### Outcomes

The questionnaire included demographics, insurance status, distance from rheumatology clinic, and detailed questions about medical and related non-medical out-of-pocket costs. Families were also asked to estimate cost of lost paid work. All questions referred to the calendar year 2009, and all costs were given in Canadian dollars. All costs that were paid by the health care system or health insurance were excluded. The following definitions were used:

1. Medical costs included costs of medications, splint material, assistive devices, and physiotherapy.

2. Non-medical costs included those costs which were not medical in nature, but were incurred in order that the child with arthritis received appropriate care. These included costs related to visits for outpatient clinics (rheumatology or other specialists, locally or at the IWK), medication infusions and hospital admissions. Costs for these visits included transportation, parking, accommodations, childcare for other children, and communications related to the visit. Non-medical costs also included those related to home adaptations and driving their child to school.

3. Finally, cost due to loss of paid work was defined as the loss of pay due to time away from work for reasons related to their child’s arthritis. This could be either or both parents.

Gross annual household income was collected to allow calculation of costs related to their child’s JIA as a proportion of the family income. Perceptions of the financial burden associated with having a child with JIA and access to financial resources were also evaluated in the questionnaire using ordered response categories.

### Analysis

Descriptive statistics were calculated for all parameters of the questionnaire. For consistency with previous literature, costs were summarized as medical, non-medical and those related to loss of paid work. The total cost was also compared to the household income per patient to determine the proportion of income lost. Spearman correlation coefficients were calculated to evaluate the relationship between costs and distance from the IWK. For this analysis, distance was estimated using travel time to the IWK, as the data was more complete than for actual distance.

The Mann–Whitney U-test was used to compare costs for between groups of children. Given the small number of patients, these analyses were considered exploratory.

## Results and discussion

Of 172 questionnaires mailed out, 5 were returned unopened due to incorrect addresses. Fifty-four (31.4%) were returned completed, and another 11 (6.6%) were returned blank or the family called to decline participation. Mothers completed 81.8% of the questionnaires.

Participant characteristics are summarized in Table 1. All JIA categories were represented and most patients were taking medications. Most patients lived at least 100 km from the IWK and/or 60 minutes away. Nearly all patients had some form of health insurance support. The perceived financial burden was rated as either large or moderate by 35.9% of respondents. Perceived access to resources to help pay for the costs was rated as poor by 36% of parents.

**Table 1:** **Characteristics of study participants** (**N** = **54**)


Age (years) (median, range)		14 (5,20)
Gender (Female: Male)		32 (59%):22 (41%)
JIA Category		
	Oligoarticular-persistent	13 (24%)
	Oligoarticular-extended	8 (15%)
	Polyarticular, RF negative	8 (15%)
	Polyarticular, RF positive	1 (2 %)
	Systemic	7 (13%)
	Enthesitis-related	8 (15%)
	Psoriatic	7 (13%)
	Undifferentiated	2 (4%)
Current Medications (N = 53)		
	No medications	14 (26%)
	NSAIDs	30 (57%)
	DMARDs	26 (49%)
	Corticosteroids	1 (2%)
	Biologics	7 (13%)
Distance from IWK Health Centre (km) (median, range) (N = 39)		35 (4, 500)
Greater than/equal to 100 km N (%)		30 (77%)
Driving time to IWK Health Centre (min) (median, range) (N = 54)		47.5 (15, 300)
Greater than/equal to 60 minutes N (%)		26 (48%)
Gross Family income (median, range)		$70000 ($2500, $175000)
Supplemental Health Insurance (N = 53)		
	Employer Plan	21 (40%)
	Personal Private	26 (49%)
	Social Assistance	1 (2%)
	None	5 (9%)
Perceived Financial Burden (N = 53)		
	Large	1 (2%)
	Moderate	18 (34.0%)
	Minimal	24 (45.3%)
	None	10 (18.9%)
Perceived Availability of Resources (N = 50)		
	Excellent	8 (16%)
	Good	14 (28%)
	Fair	10 (20%)
	Poor	18 (36%)

*JIA*, juvenile idiopathic arthritis; *IWK*, Izaak Walton Killam Health Centre.

The median number of trips to the IWK for clinic appointments in 2009, excluding trips for joint injections or medication infusions, was 4 (range 0–27). Six children had joint injections at the IWK (once for 4 children, twice for 2 children). Two children also came to the IWK for regular infusion (10 visits and 13 visits).

Data on annual costs for 2009 are presented in Table 2. The median total annual cost per patient was $619.50 (range 0-$5,535.00), which was a median of 0.7% (range 0-37%) of the household income. Non-medical costs, primarily related to visits locally and at the IWK, were the largest cost. Medical costs, primarily medications, were a relatively small component of the total costs.

**Table 2:** **Annual costs related to having a child with juvenile idiopathic arthritis** (**in Canadian Dollars**)

	**Mean (standard deviation)**	**Median (range)**
**Annual Medical Costs**		
Medication	179.30 (328)	0 (0, 1680.00)
Physiotherapy	1.72 (13)	0 (0, 93.00)
Aids/splint materials	48.92 (98)	0 (0, 350.00)
Total	229.95 (343)	122.50 (0, 1680.00)
**Annual Non**-**Medical Costs**		
IWK Visit Costs	326.91 (396)	226.25 (0, 2286.00)
Peripheral Visit Costs	116.67 (175)	50.50 (0, 1000.00)
Total Annual Visit Costs	443.58 (427)	414.75 (0, 2286.00)
Home Adaptations/Driving to School	34.17 (217)	0 (0, 1600.00)
Total	477.75 (519)	436.25 (0, 2685.00)
**Annual Loss of Paid Work**	354.81 (712)	0 (0, 2600.00)
**Total Annual Costs**	1062.50 (1227)	619.50 (0, 5535.00)
**Percent of Gross Household Income**	2.5% (5.5)	0.7% (0, 37%)

The median cost per IWK clinic visit was $45.00 (range 0-$762.00). The median cost per clinic visit outside the IWK was $15.00 (range 0-$100.00). Due to small numbers, similar results were not calculated for emergency, infusion or joint injection visits.

There was no significant correlation between distance from the IWK and annual total costs (r_s_ = 0.18, P = 0.2). There was a moderate correlation between distance from the IWK and total annual visit costs to the IWK (r_s_ = 0.30, P = 0.03).

In exploratory analyses, children with oligoarticular JIA had a median annual total cost of $359.00 (range $32-1205.00) while those with all other categories combined had a median annual total cost of $877.00 (range $0-5535.00, P = 0.02). Children living more than 60 minutes from the IWK had a mean annual total cost of $893.50 (range $0-5535.00) while those living closer than 60 minutes away had a mean annual total cost of $460.38 (range $0-3284.00, P = 0.07).

Families who rated the financial burden of having a child with JIA as being either large or moderate had a median annual total cost of $1205.00 (range $120-5036.00) compared to $474.88 (range $0-5535, P = 0.006) for those who rated it as minimal or none. The proportion of gross family income represented by the total costs of JIA were 3.0% (large or moderate burden) and 0.5% (minimal or no financial burden) (P = 0.002).

## Conclusions

This study reports the financial costs borne by families of children with JIA living in Nova Scotia in 2009. We have shown that costs vary dramatically between households. The majority of costs were related to medical visits, followed by costs related to time away from paid work. Medical costs formed a relatively small component of the total costs. We found that disease category was associated with cost, with oligoarticular JIA resulting in smaller costs than other types. We also saw a trend towards increasing costs with increasing distance from the IWK.

On average, total costs were relatively modest. However, our data show 2 important related findings. First, while costs were not large for most families, some families experienced total costs as high as 37% of their gross household income. Second, almost 36% of respondents reported their perception of the financial burden as being either moderate or large. It could be argued that having nearly 2/3 of families experiencing mild or no financial burden is a success of the health care system. However, we believe that we can do better, and our study highlights financial burden as an important issue for many families, and one that should be monitored in pediatric rheumatology clinics. Given that 36% of respondents also considered resources to assist with costs to be poor, further attention is needed to this issue.

We expected that distance from the IWK Health Centre would be an important factor impacting cost. Given the relatively widespread, rural population of Nova Scotia, this is an important issue in our clinic. However, our data showed no significant correlation between distance and total cost and only a moderate correlation between distance and IWK specific visit costs. There was a non-significant trend for families living more than 60 minutes away to have higher costs. The explanation for this lack of a relationship is unclear. It may be that some costs are unrelated to distance (including all medical costs, and some visit costs, such as parking). Additionally, it is possible that follow-up frequency is influenced by distance for children with similar JIA. It is important that all children receive optimal care and follow-up, regardless of residence. Our data was not adequate to investigate this question, but this is an issue which bears additional study. We did note that costs associated with IWK visits were higher than those associated with local visits ($45.00 vs $15.00), suggesting that distance had at least some impact.

Comparing our results to previous work has some important challenges. The costs associated with health care and the financial responsibility for these costs varies considerably between countries and between regions within countries. This makes extrapolation of research conducted in one country or region to another country or region difficult. Health care costs can also vary considerably over time, as new medications become available or health care coverage changes. For example, in JIA the advent of biologic medications has the potential to markedly influence medical costs.

Despite these limitations, some research in this area has been conducted. Allaire et al. reported costs for 70 families with a child with juvenile rheumatoid arthritis (JRA) from the New England area in 1989 [3]. Total yearly family out-of-pocket expenses were $1525 USD ($1818 CDN; all non-Canadian dollar costs converted to Canadian dollars for comparison using the mean conversion rate for year of publication or year of data collection if available). This included $708 USD for medical costs (professional services, investigations, medications and casts/splints), $488 USD for non-medical costs (transportation, meals, lodging, child-care, home adaptations) and $328 USD for loss of paid work. This total represented 5% of the annual family income. They also found that children with systemic JRA had greater costs than either the polyarticular or pauciarticular JRA groups.

Minden et al. reported on a group of 215 adults in Berlin with a history of JIA (mean age 23 years, mean disease duration 17 years) [4]. They found that out-of-pocket costs in 1999 were €77 ($117 CDN) of which €66 was medical (medications) and €11 was non-medical (transportation). They determined that the mean cost due to lost paid work was €1571. They also found that patients with rheumatoid factor positive polyarticular JIA and extended course oligoarticular JIA, as well as those with greater disease activity or functional limitations, had the highest costs. The authors point out that only 50% of these patients had seen a rheumatologist in the preceding 12 months and 1/3 were rated as having insufficient specialized care by the investigators. Given the lack of contact with care-providers, it is not clear how to interpret these results.

Bernatsky et al. compared 155 children with JIA to 181 outpatient clinic controls without JIA in Montreal and Vancouver, Canada in 2005 [5]. This study determined total direct medical costs, but did not distinguish those borne by the health-care system and those borne by the family. However, they did report an annual loss of paid work by caregivers of $1241 CDN. They also noted a direct relationship between total direct costs and disease activity.

Minden et al. studied 369 children with JIA from a national German database, with costs estimated for 2003 [6]. While this study focused on the overall economic impact of JIA on the health care system, they found that families spent €223 ($343 CDN) per year. This consisted of €54 for medical costs (medications, treatments) and €169 for non-medical costs (€132 for transportation, remainder for telephone, home alterations, childcare). They reported costs due to lost paid work to be €270 per year. Finally, as with other investigators, they found that costs varied significantly between JIA categories, with rheumatoid factor positive polyarticular JIA having the highest costs, and that costs increased with increasing disease duration, disease severity, pain, functional limitations, uveitis and delays to first pediatric rheumatology appointment.

Thornton et al. studied 297 children newly diagnosed with JIA at 4 UK hospitals [7]. While the year of study entry/diagnosis was not provided, costs were extrapolated to 2005. This study calculated primarily direct medical costs to the health care system and did not address the issue of patient or family out-of-pocket costs.

Finally, Yucel et al. studied 100 children with JIA in Turkey in 2008 [8]. Due to the nature of the health care system in Turkey at the time of the study, virtually all medical costs (medications, investigations, physicians) were not paid by families. However, the authors did find that families paid €188 ($324 CDN) per year in non-medical costs (transportation, accommodations) and an additional €81 ($140 CDN) per year in costs due to lost paid work.

In our study, we found that costs were higher than reported by most investigators, other than Allaire et al. [3]. However, costs due to loss of paid work were less than reported by other investigators (median $0, mean $354.81). These differences may reflect differences in our population, such as a more dispersed rural population or more parents engaged in unpaid work (either in the home or seasonal work such as fishing). The relationship of these differences to financial burden and the ability to achieve good care warrants further investigation.

Only 13% of patients in this study were treated with biologics. Over time, this proportion is increasing. However, the impact on costs to families is not easy to predict. Given the expense of these medications, it is unlikely that most families could afford them without insurance or other support. In our clinic, we have not prescribed biologics where families have not had some form of coverage. Even with insurance, families may have co-pay charges which could be considerable. Biologic therapy could also have an impact on visit costs, as some of the biologic treatments require infusions at the hospital, clinic or infusion centre. These evolving costs will require monitoring over time.

As with all questionnaire-based research, this study needs to be interpreted with certain limitations in mind. First, we experienced a relatively modest response rate. This is not uncommon in this type of research, but does raise questions about how well respondents represent the overall population. The JIA category distribution of respondents is similar to our overall clinic population. If there is a bias introduced here, it is difficult to predict in which direction it may operate. It is possible that some families with significant financial concerns would be more likely to respond. However, it is also possible that those same families would be less likely to respond due to concerns about being stigmatized. A second limitation is recall bias. To minimize this, a chart review was completed to supplement and confirm data from the questionnaire, especially the number of visits to the IWK. Finally, costs are specific to location and health care system. This limits the direct applicability of our results to other jurisdictions. However, our overall findings are likely to be similar to other Multidisciplinary Rheumatology Clinics that provide services to large geographic areas.

The impact of the financial burden on families of children with JIA is unknown. Beyond psychosocial stressors in the home, concern has been raised about the effect on outcomes. A prospective cohort study in a tertiary care center in the United States showed a significantly higher physical disability rate and lower health-related quality of life in children with JIA who had Medicaid or other similar state-programs for low-income families compared to those children with JIA who had insurance coverage [9]. This study suggests that it may be important to identify families who are struggling financially to optimize their child’s medical care.

We conclude that while the overall costs associated with having a child with JIA in Nova Scotia are modest for many families, the financial burden can be significant for some and may become a barrier to the provision of appropriate care. Many families perceive the financial burden to be high and the access to resources to be poor. Assistance in the form of targeted programs to aid families with the greatest costs should be considered. The impact of the financial burden on outcomes in children with JIA is unknown and warrants further evaluation.

## Abbreviations

JIA: Juvenile idiopathic arthritis; NS: Nova Scotia; IWK: Izaak Walton Killam; JRA: Juvenile rheumatoid arthritis.

## Competing interests

The authors have conflicts to declare, whether financial or otherwise.

## Author’s contributions

The project was conceived, developed and conducted by AE under the supervision of AH. BL, SR and ES contributed to project development. AE and AH conducted the analysis. All authors contributed equally to manuscript preparation and revision. All authors read and approved the final manuscript.

## Author’s information

AE (at time of study) was a resident in pediatrics at the IWK Health Centre. She is currently a fellow in pediatric endocrinology at the IWK Health Centre. AH, BL, SR and ES are pediatric rheumatologists at the IWK Health Centre.

## Supplementary Material

Additional file 1 **Appendix.** Cost of JIA Questionnaire. Click here for file
