# Supplementary material for: The financial burden of juvenile idiopathic arthritis: a Nova Scotia experience
Source: Pediatr Rheumatol Online J. 2013 May 29;11:24. doi: 10.1186/1546-0096-11-24 (PMC3711732; doi:10.1186/1546-0096-11-24)
Supplement: Additional file 1 — Appendix. Cost of JIA Questionnaire. [file 1546-0096-11-24-S1.docx]

**Appendix 1**

**Cost of JIA Questionnaire**

SECTION A

1. **How much of a financial burden is your child’s arthritis?**

None

Minimal

Moderate

Large

1. **How would you rate the resources available to help your family pay for expenses related to your child’s arthritis?**

Excellent

Good

Fair

Poor

1. **How old is your child with arthritis?**

____________ years

1. **What gender is your child with arthritis?**

Male

Female

1. **What is the relationship of the person completing this survey to your child with arthritis?**

Mother

Father

Other (please specify _____________)

1. **How long does it take to drive to the IWK from your home?**

_________ hours __________minutes

1. **How far is your home from the IWK?**

_________ kilometers

1. **In the future we may do follow-up studies looking at financial burden for families, can we contact you?**

Yes

No

Please continue on the next page

SECTION B: Costs for Medications

1. **Have you paid part or full payment for medications for your child’s arthritis in 2008?**

- No (SKIP to Section C on the next page)
- Yes

**If yes, approximately how much did you pay out-of-pocket for medication related to your child’s arthritis in 2008? Please only list the amount of money that has not been reimbursed. You may fill out one of part A (total sum in 2008) or part B (cost per medication in 2008) dependent on your personal preference.**

**Part A**

| **Total cost of medication in 2008 (including deductibles)** | **$** |
| --- | --- |

**If you have filled out Part A please proceed to SECTION C on page 3. Otherwise please fill out the chart below.**

**Part B**

| Medication | Cost per month in 2008 |
| --- | --- |
| 1. | 1. $ per month |
| 2. | 1. $ per month |
| 3. | 1. $ per month |
| 4. | 1. $ per month |
| 5. | 1. $ per month |
| 6. | 1. $ per month |
| 7. | 1. $ per month |
| 8. | 1. $ per month |
| 9. | 1. $ per month |
| 10. | 1. $ per month |

SECTION C: Costs for Aids

1. **In 2008 did your child need to use any aids because of his/her arthritis? For example splints, orthotics, wheel chairs, modified chairs, canes/crutches, built up pencils etc**.

- No (SKIP to Section D below)
- Yes

**If yes, please list the different aids your child used and the amount of money you paid out-of-pocket for each aid in 2008. Please only list the amount of money you paid out-of-pocket that was not reimbursed.**

| Aid | Cost of Aid in 2008 |
| --- | --- |
| 1. | 1. $ |
| 2. | 1. $ |
| 3. | 1. $ |
| 4. | 1. $ |
| 5. | 1. $ |

SECTION D: Cost for Physiotherapy:

1. **How much did you spend in 2008 on physiotherapy fees? Please only list the amount of money that was not reimbursed.**

$ ______________________

Please continue on the next page

SECTION E: Cost for Home Adaptations/ Lifestyle Modifications

1. **Have you had to make adaptations to your home in 2008 because of your child’s arthritis?**

- No (SKIP to Question 13)
- Yes

**If yes, what changes did you make in 2008 and how much did each adaptation cost? Please only list the amount of money you have paid out-of-pocket that was not reimbursed.**

| **Home Adaptation** | **Cost for home adaptation in 2008** |
| --- | --- |
| 1. |  |
| 2. |  |
| 3. |  |
| 4. |  |
| 5. |  |

1. **Have you had to drive your child with arthritis to school because he/she is unable to walk or take the bus to school due to their arthritis?**

- No (SKIP to Section F on next page)
- Yes

**If yes, how much on gas do you spend per week on gas driving your child to and from school?**

$_________________

Please continue on the next page

SECTION F: Costs for Joint Injections at the IWK Health Centre

1. **In 2008 did your child have any joint injections at the IWK Health Centre?**

- No (SKIP to Section G on page 7)
- Yes

1. **If yes, how many joints injection appointments did your child have in 2008?**

________________ (number of visits for joint injections)

1. **How does your child usually get to the IWK Health Centre for his/her joint injection?**

- Personal car or vehicle
- Rental car
- Bus
- Shuttle
- Train
- Taxi
- Other ( please specify ­­­­­­­­­­­_______________________)

1. **If you drive a car to your child’s joint injection appointment how much do you usually spend on parking each time?**

$________________________________

1. **If you drive a car to your child’s joint injection appointment how much do you usually spend on gas? Please give the total round-trip cost for gas.**

$________________________________

1. **If you take a taxi, bus, train or shuttle to the IWK Health Centre for your child’s joint injections how much do you usually spend for the round trip on taxi-fares or tickets? Please include the cost for your child and all individuals who travelled with your child.**

$_________________________________

1. **During a trip to the IWK Health Centre for your child’s joint injection appointment how much money do you usually spend on food? Please include the total cost for food, whether purchased at the IWK or elsewhere, for your child and all individuals who travelled with your child.**

$________________________________

1. During a visit to the IWK for a joint injection appointment, do you ever have to spend a night outside your home because it is too difficult to travel to and from the IWK in one day?

No (SKIP to Question 22)

- Yes

**If yes, how many nights have you spent away from your home in 2008 for joint injection appointments?**

**_______________________** nights away in 2008

**Please circle the one location that best describes where you and your child usually stay during a trip to Halifax for a joint injection appointment. Please list how much you spend on your accommodations per night in the right hand column?**

| **Accommodation** | **Cost per night** |
| --- | --- |
| Hotel | $ per night |
| Family or friends | $ per night |
| Ronald McDonald House | $ per night |
| Point Pleasant Lodge | $ per night |
| Other (please specify)  _______________________________ | $ per night |

1. **Do you have other children at home who require babysitting while you are at your child’s joint injection appointment?**

- No (SKIP to Question 23)
- Yes

If yes, how much do you usually spend on babysitting per joint injection visit?

$_________________________

1. **For each trip to the IWK Health Centre for a joint injection appointment, how much do you usually spend on necessary telephone calls?**

$ ________________________

SECTION G: Costs for Medical Day Unit Visits at the IWK Health Centre

1. **In 2008 did your child have any Medical Day Unit visits at the IWK Health Centre? (eg. Drug Infusions)**

- No (SKIP to Section H on page 9)
- Yes

**If yes, how many Medical Day Unit visits did your child have in 2008?**

________________ (number of Medical Day Unit visits)

1. **How does your child usually get to the Medical Day Unit at the IWK Health Centre?**

- Personal car or vehicle
- Rental car
- Bus
- Shuttle
- Train
- Taxi
- Other ( please specify ­­­­­­­­­­­_______________________)

1. **If you drive a car to your child’s Medical Day Unit visit how much do you usually spend on parking each time?**

$________________________________

1. **If you drive a car to your child’s Medical Day Unit visit how much do you usually spend on gas? Please give the total round-trip cost for gas.**

$________________________________

1. **If you take a taxi, bus, train or shuttle to your child’s Medical Day Unit visit how much do you usually spend for the round trip on taxi-fares or tickets? Please include the cost for your child and all individuals travelling with your child to his/her Medical Day Unit visit.**

$________________________________

1. **During a trip for a Medical Day Unit visit, how much money do you usually spend on food? Please include the total cost for food, whether purchased at the IWK or elsewhere, for your child and all individuals who travelled with your child.**

$________________________________

1. **During a visit to the Medical Day Unit, do you ever have to spend a night outside your home because it is too difficult to travel to and from the IWK in one day?**

No (SKIP to Question 31)

Yes

**If yes, how many nights did you spend away from your home in 2008 because Medical Day Unit visits for your child’s arthritis?**

**_________________** nights away in 2008

**Please circle the one location that best describes where you and your child usually stay during a trip to Halifax for a Medical Day Unit visit. Please list how much you spend on your accommodations per night in the right hand column?**

| **Accommodation** | **Cost per night** |
| --- | --- |
| Hotel | $ per night |
| Family or friends | $ per night |
| Ronald McDonald House | $ per night |
| Point Pleasant Lodge | $ per night |
| Other (please specify)  _______________________________ | $ per night |

1. **Do you have other children at home who require babysitting while you are at a Medical Day Unit appointment for your child’s arthritis?**

- No (SKIP to Question 32)
- Yes

If yes, how much do you usually spend on babysitting per Medical Day Unit visit?

$_________________________

1. **For each trip to the Medical Day Unit how much do you usually spend on necessary telephone calls?**

$ ________________________

SECTION H: Costs for Appointments at the IWK Health Centre

1. **In 2008 how many trips did your child have to make to the IWK Health Centre for appointments related to his/her arthritis? This would include appointments to see the Rheumatologist, Nephrologist, Ophthalmologist, Physiotherapy etc. If your child made one trip to see multiple physicians or health care providers during the same visit please only count it as one trip. Please do NOT include trips for joint injections or visits to the Medical Day Unit.**

_______________ trips to the IWK in 2008

1. **How does your child usually get to their appointments at the IWK Health Centre?**

- Personal car or vehicle
- Rental car
- Bus
- Shuttle
- Train
- Taxi
- Other ( please specify ­­­­­­­­­­­_______________________)

1. **If you drive a car to your child’s appointments how much do you usually spend on parking at the IWK per visit?**

$______________________

1. **If you drive a car to your child’s appointments how much do you usually spend on gas? Please give the total round-trip cost for gas.**

$**______________________**

1. **If you take a taxi, bus, train or shuttle to your child’s appointments at the IWK Health Centre how much do you usually spend for the round trip on taxi-fares or tickets? Please include the cost for your child and all individuals travelling with your child to the IWK Health Centre.**

$_____________

1. **During a trip to the IWK Health Centre how much money do you usually spend on food? Please include the total cost of food, whether purchased at the IWK or elsewhere, for your child and all individuals who travelled with your child to his/her appointment.**

$_________________________

1. During a visit to the IWK, do you have to spend a night outside your home because it is too difficult to travel to and from the IWK in one day?

No (SKIP to Question 40)

- Yes

**If yes, how many nights have you spent away from your home in 2008 for appointments at the IWK?**

**_______________________** nights away in 2008

**Please circle the one location that best describes where you and your child usually stay during a trip to Halifax for an appointment related to your child’s arthritis. Please list how much you spend on your accommodations per night in the right hand column?**

| **Accommodation** | **Cost per night** |
| --- | --- |
| Hotel | $ per night |
| Family or friends | $ per night |
| Ronald McDonald House | $ per night |
| Point Pleasant Lodge | $ per night |
| Other (please specify)  _______________________________ | $ per night |

1. **Do you have other children at home who require babysitting while you are at an appointment at the IWK Health Centre for your child’s arthritis?**

- No (SKIP to Question 41)
- Yes

If yes, how much do you usually spend on babysitting per appointment visit?

$_________________________

1. **For each trip to the IWK Health Centre for an appointment for your child’s arthritis, how much do you usually spend on necessary telephone calls?**

$ ________________________

SECTION I: Costs for Appointments at your local hospital, community health centre or doctor’s office

1. **In 2008 how many visits did your child have with doctors or health professionals at your local hospital, community health centre or a doctor’s office because of issues related to his/her arthritis? This may include visits to the Ophthalmologist, Physiotherapist, Occupational Therapist, bloodwork etc. If your child saw more than one doctor or health professional during a visit please count that visit as only one visit.**

__________________ visits in 2008

1. **Approximately how much does it cost per visit to, your local hospital, community health care centre, or doctor’s office? Please include transportation, parking, and food costs?**

$ _______________ per visit

Please continue on the next page

SECTION J: Emergency Room Visits at the IWK Health Centre

1. **In 2008 did you take your child to the IWK Emergency Department for reasons related to his or her arthritis?**

- No (SKIP to Section K on page 14)
- Yes

1. **In 2008 how many visits did your child have to the emergency department at the IWK Health Centre for reasons related to his/her arthritis.**

___________________ visits

1. **How does your child usually get to the emergency department at the IWK Health Centre?**

- Personal car or vehicle
- Rental car
- Bus
- Shuttle
- Train
- Taxi
- Other ( please specify ­­­­­­­­­­­_______________________)

1. **If you drive a car to the IWK emergency department how much do you usually spend on parking per visit?**

$______________________

1. **If you drive a car to the emergency department how much did you usually spend on gas? Please give the total round-trip cost for gas.**

$**______________________**

1. **If you take a taxi, bus, train or shuttle to the emergency department at the IWK Health Centre how much did you usually spend for the round trip on taxi-fares or tickets? Please include the cost for your child and all individuals travelling with your child to the IWK Health Centre.**

$_____________

1. **During a visit to the IWK Emergency Department in 2008 how much money do you usually spend on food? Please include the total cost of food, whether purchased at the IWK or elsewhere, for your child and all individuals who travelled with your child to his/her appointment.**

$_________________________

1. During a visit to the IWK Emergency Department in 2008, did you have to spend a night outside your home because it is too difficult to travel to and from the IWK in one day?

No (SKIP to Question 52)

- Yes

**If yes, how many nights have you spent away from your home in 2008 for visits to the IWK Emergency Department?**

**_______________________** nights away in 2008

**Please circle the one location that best describes where you and your child usually stayed during a trip to Halifax for a visit to the IWK Emergency Department because of reasons related to your child’s arthritis. Please list how much you spent on your accommodations per night in the right hand column?**

| **Accommodation** | **Cost per night** |
| --- | --- |
| Hotel | $ per night |
| Family or friends | $ per night |
| Ronald McDonald House | $ per night |
| Point Pleasant Lodge | $ per night |
| Other (please specify)  _______________________________ | $ per night |

1. **Do you have other children at home who required babysitting while you were at the IWK Emergency Department for your child’s arthritis?**

- No (SKIP to Question 53)
- Yes

If yes, how much do you usually spend on babysitting per appointment visit?

$_________________________

1. **In 2008 for each trip to the IWK Emergency Department for reasons related to your child’s arthritis, how much did you usually spend on necessary telephone calls?**

$ ________________________

SECTION K: Emergency Room Visits at Local Hospitals

1. **In 2008 did you take your child to a local emergency department for reasons related to his or her arthritis?**

- No (SKIP to Section L on page 16)
- Yes

1. **In 2008 how many visits did your child have to your local emergency department for reasons related to his/her arthritis.**

___________________ visits

1. **How did your child usually get to the emergency department at your local hospital?**

- Personal car or vehicle
- Rental car
- Bus
- Shuttle
- Train
- Taxi
- Other ( please specify ­­­­­­­­­­­_______________________)

1. **If you drive a car to your local emergency department how much did you usually spend on parking per visit?**

$______________________

1. **If you drive a car to your local emergency department how much did you usually spend on gas? Please give the total round-trip cost for gas.**

$**______________________**

1. **If you take a taxi, bus, train or shuttle to the emergency department at your local hospital how much did you usually spend for the round trip on taxi-fares or tickets? Please include the cost for your child and all individuals travelling with your child to the emergency department.**

$______________________

1. **During a visit to your local emergency department in 2008 how much money did you usually spend on food? Please include the total cost of food, whether purchased at the hospital or elsewhere, for your child and all individuals who travelled with your child to his/her appointment.**

$_________________________

1. During a visit to your local emergency department in 2008, did you have to spend a night outside your home because it was too difficult to travel to and from the hospital in one day?

No (SKIP to Question 62)

- Yes

**If yes, how many nights have you spent away from your home in 2008 for visits to the local emergency department?**

**_______________________** nights away in 2008

**Please circle the one location that best describes where you and your child usually stayed during a trip to your local emergency department because of reasons related to your child’s arthritis. Please list how much you spent on your accommodations per night in the right hand column?**

| **Accommodation** | **Cost per night** |
| --- | --- |
| Hotel | $ per night |
| Family or friends | $ per night |
| Ronald McDonald House | $ per night |
| Point Pleasant Lodge | $ per night |
| Other (please specify)  _______________________________ | $ per night |

1. **Do you have other children at home who required babysitting while you were at your local emergency department because of your child’s arthritis?**

- No (SKIP to Question 63)
- Yes

If yes, how much do you usually spend on babysitting per visit?

$_________________________

1. **In 2008 for each trip to your local emergency department for reasons related to your child’s arthritis, how much did you usually spend on necessary telephone calls?**

$ ________________________

SECTION L: Costs for Admissions to IWK Health Centre

1. **In 2008 was your child admitted to the IWK Health Centre for reasons related to his/her arthritis? Please do not include admissions to other hospitals.**

- No (SKIP to Section M on page 18)
- Yes

1. **If yes, how many days has your child spent admitted to the IWK Health Centre for reasons related to his/her arthritis in 2008?**

**____________** days

1. **While your child was admitted to the IWK Health Centre what form of transportation did you usually use to get to and from the hospital?**

- Personal car or vehicle
- Rental car
- Bus
- Shuttle
- Train
- Taxi
- Other ( please specify ­­­­­­­­­­­_______________________)

1. **If you drove a car, during your child’s admission to the IWK Health Centre how much did you usually spend per day on parking?**

$_____________ per day

1. **If you drove a car, during your child’s admission to the IWK Health Centre how much did you usually spend per day on gas travelling to and from the hospital?**

$_____________ per day

1. **If you took a bus, train, shuttle or taxi, during your child’s admission to the IWK Health Centre how much did you spend per day on tickets or taxi-fares?**

$_____________ per day

1. How much did your immediate family usually spend per day on food eaten at the hospital?

$_____________ per day

1. **Do you have other children at home who required babysitting while your child was admitted to the IWK Health Centre for reasons related to his/her arthritis?**

- No (SKIP to Question 72)
- Yes

If yes, how much did you usually spend per day on babysitting?

$_____________ per day

1. **During your child’s admission(s), how much did you usually spend on telephone calls and cable per day?**

$______________ per day

SECTION M: Costs for Admissions to Other Hospitals

1. **In 2008 was your child admitted to a hospital other than the IWK for reasons related to his/her arthritis? Please do not include admissions to other hospitals.**

- No (SKIP to Section N on page 19 half way down the page)
- Yes

1. **If yes, how many days in 2008 did your child spend admitted to your local hospital for reasons related to his/her arthritis?**

**____________** days

1. **While your child was admitted to the hospital what form of transportation did you usually use to get to and from the hospital?**

- Personal car or vehicle
- Rental car
- Bus
- Shuttle
- Train
- Taxi
- Other ( please specify ­­­­­­­­­­­_______________________)

1. **If you drove a car during your child’s admission how much did you usually spend per day on parking?**

$_____________ per day

1. **If you drove a car, during your child’s admission how much did you usually spend per day on gas travelling to and from the hospital?**

$_____________ per day

1. **If you took a bus, train, shuttle or taxi, during your child’s admission how much did you spend per day on tickets or taxi-fares?**

$_____________ per day

1. How much did your immediate family usually spend per day on food eaten at the hospital?

$_____________ per day

1. **Do you have other children at home who required babysitting while your child was admitted to the hospital for reasons related to his/her arthritis?**

- No (SKIP to Question 81)
- Yes

If yes, how much did you usually spend per day on babysitting?

$_____________ per day

1. **During your child’s admission(s), how much did you usually spend on telephone calls and cable per day?**

$______________ per day

Please continue on the next page

SECTION N: Loss of Paid Work

1. **In 2008 have you taken time off of paid work for appointments, admissions or other reasons related to your child’s arthritis?**

- No (SKIP to Question 83)
- Yes

If yes, about how much money have you lost because of missed employment in 2008 due to reasons related to your child’s arthritis?

$_____________________

1. In 2008 have other adults in your household taken time off of paid work for appointments, admissions or other reasons related to your child’s arthritis? Please include only adults who live in the same residence as your child with arthritis.

- No (SKIP to Section O at the bottom of the page)
- Yes

1. **If yes, how is this person related to your child with arthritis?**

Mother

Father

Other (please specify _____________)

1. About how much money has he/she lost because of missed employment in 2008 due to reasons related to your child’s arthritis?

$__________________

SECTION O: Sources of Income

**The following section asks questions about your family’s income. As further outlined in the consent document, all information will be kept in the strictest confidence. We are asking for this information so we can compare annual costs to family income.**

1. **According to your 2008 tax return how much was your gross household income? Please include all wage earners living in your home. If you do not want to answer this question please check the box stating you have chosen not to answer.**

| ⁯ $0-5,000 | ⁯ $40,001-50,000 | ⁯ $150,001-200,000 |  |
| --- | --- | --- | --- |
| ⁯ $5,001-10,000 | ⁯ $50,001-60,000 | ⁯ $200,001-300,000 |  |
| ⁯ $10,001-15,000 | ⁯ $60,001-80,000 | ⁯ $300,001-400,000 |  |
| ⁯ $15,001-20,000 | ⁯ $80,001-100,000 | ⁯ $400,001-500,000 |  |
| ⁯ $20,001-30,000 | ⁯ $100,001-120,000 | ⁯ $500,001-800,000 |  |
| ⁯ $30,001-40,000 | ⁯ $120,001-150,000 | ⁯ > $800,000 |  |

- I do not want to answer this question

**Please continue on the next page**

1. **Do you have medical coverage from any of the following insurance plans?**

- Private health insurance plan (ex. Blue Cross)
- Social assistance health insurance plan
- Employer health insurance plan
- We do not have a health insurance plan
- I do not know

1. **Have you received financial assistance from any of the following resources? Please check all that apply**

- Community Fundraising
- Parent living outside your household
- Other family members or friends
- IWK Social Work
- Social Services
- Other ( if so please specify: ______________________________________)

1. **How much money did the resources listed in question 87 contribute in 2008?**

Community Fundraising $__________________________

Parent living outside your household $__________________________

Other family members or friends $__________________________

IWK Social Work $__________________________

Social Services $__________________________

Other $__________________________

1. **What would be useful to help you with the expenses associated with your child’s arthritis?**

______________________________________________________________________________________________________________________________________________________________________________________________________________________________________________________________________________________________________________________________________________________________________________________________________

Thank you for completing this questionnaire.
